# Culturally and Linguistically Diverse Gamblers of East Asian Descent in Australia: A Comprehensive Review of Current Evidence

**DOI:** 10.1007/s10899-023-10202-5

**Published:** 2023-03-28

**Authors:** Victoria Rowlatt, Darren Wraith, Thuy-Vi Minh Doan, Christina Malatzky

**Affiliations:** School of Public Health and Social Work, Queensland University of Technology, Brisbane, QLD Australia

**Keywords:** Gambling, Casino, Culture, Asian, Help-seeking, Culturally and linguistically diverse

## Abstract

As a country with one of the highest per capita gambling losses per year in the world, and an evolving multicultural profile, Australia has become an important setting in which to examine the harms and benefits related to gambling. The Australian population includes people from East Asian cultural backgrounds who are a key demographic of interest for gambling operators planning to grow revenue. However, Australian gambling research has concentrated primarily on those belonging to the dominant cultural group. Most of the previous and limited number of studies to examine gambling among culturally and linguistically diverse (CALD) residents have focused on people of Chinese descent, and much of the literature is now becoming relatively old. This review examines the current evidence around cultural variations in gambling prevalence, motivations, beliefs, behaviours, and help service utilisation, focusing on gamblers with an East Asian cultural background. Numerous domains in which gambling motivations and behaviours vary across cultural groups are identified, and methodological considerations related to ethnographic gambling research are discussed. This review found that while barriers and predictors to help-seeking for CALD gamblers have been studied extensively, contemporary evidence of help service utilisation and effectiveness in Australia is lacking. Further research providing an accurate assessment of the impacts of gambling for CALD gamblers is needed to ensure that harm minimisation resources are effective for those most vulnerable to harm.

**Supplementary Information:**

The online version contains supplementary material available at 10.1007/s10899-023-10202-5.

The availability of gambling activities has increased substantially in recent years, and the World Health Organization now recognises gambling-related harm as a significant public health issue (Abbott, 2020). This is particularly evident in Australia, where gambling is easily accessible in clubs, hotels, and online, and many major Australian cities host large destination casinos. Australia has the highest gambling losses per capita in the world. People living in Australia spend 2.023% of their household disposable income on gambling (Badji et al., 2020; Queensland Government Statistician’s Office, 2021). In just one Australian state, Queensland, over 45,000 electronic gaming machines (EGMs) are distributed across the 1,116 hotels and clubs with gaming licences. Given this environment, Australia has become an important setting in which to examine the harms and potential benefits related to gambling (Abbott et al., 2015; Delfabbro & King, 2012; Farrell & Fry, 2021; Hilbrecht et al., 2020; Marko et al., 2022; Tulloch et al., 2021).

Australia is also a country with a relatively unique and evolving cultural profile. Currently, 51.5% of people residing in Australia were either born overseas or have parents who were born overseas (Australian Bureau of Statistics, 2021). Communities from East Asian countries or raised by family members from these countries represent a substantial proportion of the population. Nationally, 10.9% of the population identify as having Eastern Asian ancestry, and 7.5% were born in an East Asian country. A similar demographic profile is found in Queensland (Australian Bureau of Statistics, 2021). Despite this, gambling research has concentrated primarily on the gambling behaviours, experiences of, and implications for those belonging to the dominant cultural group within Western countries like Australia. Of those studies that have examined gambling among culturally and linguistically diverse (CALD) residents, most have focused on people of Chinese descent (Blaszczynski et al., 1998; Chan et al., 2015; Chee & Lui, 2021; Lam, 2005; Loo et al., 2008; Oei & Raylu, 2007, 2009, 2010). Relatively little research engages with people belonging to other East Asian cultural groups.

The meaning of culture and its application in gambling research can be interpreted in various ways. Guided by the work of other researchers in the field (Causadias et al., 2021; Guitart & Ratner, 2011; Raylu & Oei, 2004), we conceptualise culture as the complex interaction between people, environment, and practices that are socially inherited within a group at the macro level and influence its members at the micro level. Culture is expressed in individuals’ thoughts, values, behaviours, and personal definitions of self. The meaning given to gambling is inevitably influenced by the cultural system within which individuals are located (Abt et al., 1985). However, culture is a dynamic construct that is subject to both top-down and bottom-up processes of change (Erez & Gati, 2004). In this review, it is not our intention to imply that belonging to any particular cultural group constitutes a risk factor in and of itself. Rather, we seek to draw into focus the various ways cultural groups perceive gambling, which can impact individual behaviours, and help service needs.

Gambling research focused on cultural groups has historically emphasised differences between individualist cultures, understood to be common in predominately Western societies, and collectivist cultures, which characterise many societies in Asia. Differences between these two cultural systems span multiple domains, including across the cognitive and motivational processes of individuals, in systems of values and meaning, and in the sensitivity to and expression of social norms (Aliyev & Wagner, 2018; Balcetis et al., 2008; Hagger et al., 2014; Mesquita, 2001). Changes in the cultural makeup of societies over time influence broader cultural systems and group behaviours as well as individual behaviours (Brewer et al., 2013; Noble & Ang, 2018; Raymer et al., 2018; van Oudenhoven & Ward, 2013). There is great potential to extend the field of gambling studies by investigating how specific cultural ideas and norms influence gambling behaviours. However, as Dickens and Thomas (2016) argue, much of the literature related to cultural variations in gambling behaviours to date is relatively old. New research is needed to determine if previously found trends remain relevant. This is especially applicable in the Australian context, given the country’s changing cultural profile.

## The Case of Australia

Alongside East Asian individuals born overseas are Asian-born Australians positioned within and influenced by East Asian and westernised cultural systems. Given the influence of both cultural systems during the formative years and into adulthood, the gambling behaviours of and related harms for these community members may differ from those experienced by migrant populations primarily raised in Asia. Further, it is known that Asian-born Australians with East Asian cultural backgrounds are a key target for gambling advertisements by casino operators, with market share for this demographic expected to increase significantly over the next five to ten years (The Star Entertainment Group Ltd., 2018, 2019; Tse et al., 2012). However, much of the existing research has focused on international students, immigrants experiencing the process of acculturation, the elderly, and people from East Asia who gambled within their country of origin (Chen & Dong, 2015; Oei & Raylu, 2010; Quinlan et al., 2014; Radermacher et al., 2016).

Recent investigations into Australia’s two largest casino licence operators (Crown Resorts and Star Entertainment Group) suggest that Asian-born Australians and gamblers from East Asian countries have been of great interest to gambling providers. Since 2019, numerous media reports have made allegations about casino operator Crown Resorts regarding money laundering and illegally promoting gambling by international VIP patrons of East Asian descent. This public scrutiny culminated in the Bergin Inquiry, an investigation into Crown’s suitability to hold its Sydney casino licence. The inquiry found that the casino had facilitated practices that were either illegal or in opposition to responsible gambling codes of practice (State of NSW, 2021a, 2021b). The findings of the Bergin Inquiry ultimately led to the establishment of two Royal Commissions, special public inquiries with broad powers to collect evidence, which further investigated Crown Resorts’ suitability to hold a casino licence at its Perth and Melbourne casinos. The Star Entertainment Group, the licence holder for two casinos in Queensland and another in Sydney, has also come under recent government scrutiny. A smaller external review conducted to assess its responsible gambling practices and compliance with regulators (Bell, 2022a, 2022b, 2022c; Department of Justice and Attorney-General, 2022) found evidence of practices similar to those occurring at Crown casinos.

Many illegal and harm-causing practices were investigated during the Royal Commission inquiries into Crown and the investigation into Star. These included incentivising gambling among known at-risk gamblers, promoting gambling in East Asian countries where it is illegal, the use of junkets to subvert gambling laws and codes of practice, enabling gambling by patrons linked to organised crime in Asia, and facilitating illegal transfers of money for VIP gambling among international patrons (Bell, 2022a, 2022b, 2022c; Department of Justice and Attorney-General, 2022; Perth Casino Royal Commission, 2022; State of NSW, 2021a, 2021b; The Royal Commission into the Casino Operator and Licence, 2021). The inquiries also found that, in general, substantial money laundering was occurring at casinos in multiple Australian states. Expert testimony from one law enforcement officer indicated that money laundering within casinos is common among young East Asian-identified men (The Royal Commission into the Casino Operator and Licence, 2021). There is also evidence that loansharking is a particular challenge in the context of casinos, especially regarding gamblers of East Asian descent (Blaszczynski et al., 1998; Le & Gilding, 2016; South Australian Centre for Economic Studies, 2015). Several testimonies during the Royal Commission inquiries pointed to Crown staff taking insufficient action to prevent illegal activities, including loansharking in its Melbourne casino (The Royal Commission into the Casino Operator and Licence, 2021), and that it continued despite interventions at the Perth casino (Perth Casino Royal Commission, 2022).

These investigations into casinos across multiple Australian states highlighted deficits in casino operators’ organisational culture regarding harm minimisation and responsible gambling measures. Many breaches identified during the investigations related to VIP gamblers from East Asian countries or East Asian-identified cultural groups within the casino environment. Combined, these findings highlight the importance of this demographic to the ongoing economic viability of casinos in Australia, as perceived by Australian casino operators.

In what follows, we review the extent, nature, and impact of gambling-related harms experienced by CALD gamblers of East Asian descent in Australia. Gambling-related harms affect individual gamblers and people in their immediate familial and social spheres (Goodwin et al., 2017). These harms cause social disconnection, family unit dysfunction, and financial and mental health problems for those affected (Li et al., 2016; Sobrun-Maharaj et al., 2013) and may be exaggerated in close-knit communities. With this context in mind, the purpose of this review is to provide an overview of the current knowledge and, by doing so, identify gaps and highlight key issues of concern for communities and subsequent areas for future research.

## Methodology

This review was guided by the strategies found in Snyder (2019), Onwuegbuzie and Frels (2016), and Templier and Paré (2015). Some of the authors of this review have substantive experience in gambling research, including assessing gambling behaviours and outcomes through large surveys, as well as the community impacts of gambling. Over several years, these authors have consulted extensively with gambling industry stakeholders, other gambling research experts, government bodies, and gambling help service providers. These varied collaborative relationships have allowed the authors to develop a broad knowledge of gambling, gambling research, and the Australian gambling environment. This degree of topical expertise, along with specific discipline training in the fields of psychology, public health, and sociology, underpins their positioning within this sphere and the approach to this review. Several broad key concepts were identified by using this previous knowledge and guided by the current topical interest in casino gambling in Australia. These included gambling among CALD groups, gambling among Asian gamblers, casino gambling, gambling-related harm, and gambling help service utilisation. These key concepts informed the creation of search parameters designed to retrieve relevant literature. The search parameters were then entered into online databases, including PubMed, Medline, APA PsychINFO, Embase, SpringerLink, Web of Science, and Scopus. After excluding articles that were not written in English, peer-reviewed, or on topic, over 700 results were returned.

Preliminary screening based on titles and abstracts excluded 201 articles deemed irrelevant for the current review. Literature focusing on CALD gamblers not of East Asian descent, on gambling industry employees, or on gambling that was not within a club, hotel, casino, online, or for money was also excluded. Of the remaining literature, 218 articles were deemed potentially relevant or capable of providing a wider context. These articles related to gambling prevalence, participation, harm or help service utilisation specifically focused on CALD gamblers of East Asian descent or included East Asian gamblers as a specific sub-group. Of these articles, 19 related to casino gambling and East Asian gamblers. Only 12 articles were published in the last 10 years. The 218 articles with potentially relevant information were read in-depth, and related content was coded. For example, articles related to cultural harm were given the code HARM-CUL to allow all content about this idea across different sources to be indexed and collated. Annotations on methodology and findings were made and synthesised for each article in an annotated bibliography.

During this process, much of the literature was identified as relatively old, with samples that were either very narrow or broad in relation to ethnicity inclusion criteria (e.g., only assessing Chinese gamblers or combining many Asian cultural groups) or had limited applicability to the Australian context. Other potentially relevant literature cited in included articles was also traced at this stage and incorporated into the annotated bibliography. Finally, Australian government and stakeholder reports were searched for relevant information on gambling and gambling support service utilisation among people of East Asian descent. Reports published in New Zealand and the United Kingdom were also included in the search as these countries have similar gambling environments and regulations to Australia. All relevant literature was then synthesised and analysed to produce the current review.

As part of this review, a stakeholder scan of help services that assist gamblers from CALD groups, specifically those of East Asian descent, as well as general help services in Australia, was conducted (See Table A1 in Supplementary materials). This was achieved through several methods, including internet searching, utilising existing contact networks, and snowballing.

## Gambling Participation and Problem Gambling Prevalence Within East Asian Communities

Despite heavy regulation of gambling in many East Asian countries and the conflict with the traditional ideals of Confucianism (Fan et al., 2021), some forms of gambling have a rich and socially accepted history. For example, Mah-jong and family games of cards and dice at celebrations have long been a part of Chinese culture (Arthur et al., 2008; Lai, 2006; Zheng et al., 2009). Some studies found that, despite social gambling at family events being commonplace in many Asian cultures (Scull & Woolcock, 2005), there is a lower overall participation rate in gambling among people of East Asian descent (Kim, 2012; Welte et al., 2014). A cross-cultural study by Oei and Raylu (2010) found that 17.6% of Chinese participants had never engaged in gambling compared to only 4.2% of respondents of European origin. However, it is important to note that gambling engagement and related practices vary across Asian cultures (Kim, 2012). For example, a recent prevalence study of college students in the United States found that respondents of Chinese and Vietnamese descent did not have significantly higher odds of having gambled in the past three months compared to respondents of European origin (Wong & Wu, 2020). However, students from ‘Other Asian’ countries were found to have higher odds.

Despite the evidence generally indicating lower overall participation in gambling within East Asian communities, the prevalence of problem gambling among those who do gamble is significantly higher than in other groups (Fiedor & Seidlová, 2022; Forrest & Wardle, 2011; Victorian Casino and Gaming Authority, 2000; Wong & Wu, 2020). Problem gambling rates range from 2.8% (Po Oei et al., 2008) to 10.7% in Chinese-speaking samples (Victorian Casino and Gaming Authority, 2000). However, a meta-analysis by Loo et al. (2008) found more conservative estimates of 2.4–4%. In comparison, problem gambling rates in the general Australian community tend to be lower, from approximately 1–1.5% (Armstrong & Carroll, 2017; Markham et al., 2017; Victorian Casino and Gaming Authority, 2000). An even lower estimate of 0.51% was found in the most recent wave of the Queensland Household Gambling Survey (Department of Justice and Attorney-General, 2018). High prevalence rates of problem gambling within East Asian communities have also been highlighted as an issue in the ageing population, where older East Asian gamblers are more than twice as likely to have a gambling problem compared to older European-origin gamblers (Raylu & Oei, 2004).

## Risk Factors for Problematic Gambling Among East Asian Gamblers

### Personal Cognitions

Some problem gambling risk factors, including cognitive distortions, are consistent across cultural groups (Johansson et al., 2009; Subramaniam et al., 2017; Toneatto, 1999). Higher cognitive distortion scores are significantly related to higher levels of problematic gambling, even when co-morbid psychiatric disorders and genetic and environmental influences are accounted for (Xian et al., 2008). For instance, Gaboury and Ladouceur (1989, as cited in Po Oei et al., 2008) utilised a ‘thinking aloud’ method to assess the cognitions of gamblers while gambling. They found that 70% of gamblers’ vocalisations were inaccurate. Faulty cognitions for gamblers include concepts such as the gambler’s fallacy, whereby the gambler believes that a win will follow a series of losses; flawed availability heuristics, where a gambler can more easily recall wins rather than losses; and faulty cognitions around illusions of control, where the gambler believes they have a measure of control over gambling outcomes (Ji et al., 2015; Ladouceur, 2004; Oei & Raylu, 2007; Subramaniam et al., 2017). Some evidence contradicts this for low-skill gambling activities (Moritz et al., 2021).

However, cultural factors influence how these global cognitive distortions are manifested. For example, in tasks of luck, research has found that East Asian gamblers are generally more susceptible to the gambler’s fallacy compared to gamblers of European origin (Ji et al., 2015). Chinese gamblers are more likely to endorse a positive recency fallacy (that the next outcome is likely to be the same as the previous outcome), in contrast to Westerners. They tend towards a negative recency fallacy (that the next outcome is likely different from the previous outcome) (Fong et al., 2013). Investigation of how cognitive distortions vary between cultures is important because these can impact decisions to continue gambling and may help provide insight into the discrepancy in prevalence rates.

Turning to a related concept, in contrast to Western cultures, superstitious thinking is a more common pattern in many Asian cultures (Chinchanachokchai & Chinchanachokchai, 2021; Chinchanachokchai et al., 2017; Rehm et al., 2018; Tsang, 2004) and plays a particularly strong role in the way that East Asian gamblers interpret luck and winning. While heightened perceptions of personal luck appear to be common among problematic gamblers compared to recreational gamblers (Wohl et al., 2007), an Australian sample found that Chinese gamblers were more superstitious than European-origin gamblers (Po Oei et al., 2008). Superstitions have also been found to be significantly higher in Chinese problem gamblers compared to non-problem gamblers (Ohtsuka & Chan, 2010). Superstitious thinking can impact upon illusions of control – believing that certain numbers, colours, actions, and objects can influence gambling outcomes (Chee & Lui, 2021; Ho et al., 2019; Oei & Raylu, 2007). These faulty cognitions have consistently been found to relate to problematic gambling (Myrseth et al., 2010; Po Oei et al., 2008; Subramaniam et al., 2017). This has been evidenced in young Chinese pathological gamblers, who, in Yu and Fu’s (2014) study, tended to endorse the belief that even in games of chance, there is an ability to manipulate game outcomes using willpower beyond just associating game outcomes with personal luck.

### Situational Factors

Other risk factors for problematic gambling relate to situational factors. For example, difficulties encountered during the process of acculturation, such as problems adapting to the host culture, discrimination, and isolation resulting from language barriers, have been identified as risk factors in the development of gambling problems among some CALD communities (Chee & Lui, 2021). However, evidence around the effect of acculturation is complex. Acculturation can pose a risk when migrants from countries with limited access to gambling are exposed to an environment where gambling is widely accepted and available. Some studies indicate that having resided for longer in a host country with higher gambling availability, such as the United States, is associated with older Chinese adults gambling more frequently (Chen & Dong, 2015). A higher gambling frequency can lead to an increased risk of problem gambling due to exposure (Raylu & Oei, 2004).

Despite the risks associated with increased exposure to gambling, successful adaptation to a host country can be a protective factor against gambling problems (Oei & Raylu, 2009), with cultural shifts and cultural incorporation negatively predicting problem gambling. Oei and Raylu (2009) found that cultural resistance, where one remains most strongly influenced by their culture of origin, was not a significant predictor of gambling problems. Instead, they proposed that particular negative psychological states and experiences that can occur during the acculturation process (acculturative stressors) are associated with an increased risk of problematic gambling. Examples of these stressors include loneliness, boredom, experiences of racism, or uncertainty about one’s place. However, some evidence suggests that only a small proportion of migrants who experience acculturative stressors go on to engage with gambling as a coping mechanism (Ellenbogen et al., 2007). Acculturative stress as a motivator for engaging in gambling may not be as commonplace as once thought (Ellenbogen et al., 2007). However, the impacts of acculturative stress for those who do utilise gambling in this way (Sam & Berry, 2016) have been linked to the development of gambling problems (Po Oei et al., 2008; Raylu & Oei, 2002, 2004), along with greater gambling severity and motivation to gamble (Jacoby et al., 2013).

Many casinos in Australia offer culturally specific décor, food, entertainment, and communications designed to appeal to people from CALD communities, particularly those of East Asian origin (Dickens & Thomas, 2016). There is evidence that casinos are perceived as an accepting and welcoming environment by people from East Asian cultural groups (Tse et al., 2012), where being immersed in surroundings that are reminiscent of their own cultural background and in the presence of others who belong to their cultural community can provide a sense of comfort (Raylu & Oei, 2004; Victorian Casino and Gaming Authority, 2000). Proponents of attention restoration theory have found that casinos provide a restorative space for gamblers, allowing for a break from concentrated, directed attention towards challenging or unpleasant stimuli (Rosenbaum & Wong, 2015). Therefore, the gambling environment is even more attractive to those experiencing acculturative stress. This further highlights the potential for harm in groups that frequent casinos to gamble as, compared to other gamblers, casino gamblers are more likely to be problem gamblers and engage in binge gambling (South Australian Centre for Economic Studies, 2015).

Other social and environmental factors beyond acculturation can impact the initiation of gambling and the development of gambling problems (Wu et al., 2021). While still relatively prevalent among East Asian gamblers (43%), Clarke et al. (2006) found that being introduced to gambling by family and friends was less prevalent when compared to other ethnic groups. However, having family members with current or previous gambling problems has been associated with an increased risk of developing problematic gambling through normalisation, environmental influences, and genetic discrepancies in the dopaminergic and serotonergic systems, as has been identified during twin studies (Dowling et al., 2016, 2018; Gyollai et al., 2014; Lalande et al., 2013; Williams et al., 2022). This same trend has also been found with East Asian gamblers (Oei & Raylu, 2007; Teo et al., 2007). It is possible that, despite lower overall gambling engagement within some Asian communities, gambling problems may be more readily transferrable within family and social circles due to the high prevalence of problematic gambling among those who do gamble.

## Motivations for Gambling Among East Asian Gamblers

People’s motivations to gamble evolve (Flack & Morris, 2016) and vary in diverse ways. Motivations are influenced by personal, social, and environmental factors (Lee et al., 2015). Differences in motivations have been identified between gamblers from different cultural locations. Social interaction, for example, has emerged as a significant theme in the initiation and maintenance of gambling participation for East Asian gamblers (Oei & Raylu, 2010; Tse et al., 2012) and has been described as a perceived benefit of gambling, along with positive changes in affect, escapism, and, occasionally, financial gain (Loroz, 2004; Tagoe et al., 2018; Wickwire et al., 2007). This is especially true for casino gambling (Scull & Woolcock, 2005). However, much of the research in this context relates to gamblers who are lacking in other social supports, such as international students, immigrants, and people with limited language skills. It is unclear if Asian-born Australians with established social supports are motivated by the opportunity for social interaction that is provided by gambling, especially at casinos.

The Gambling Motivations Scale (Chantal et al., 1994), which has been validated for use with people of Chinese descent (Wu & Tang, 2010), identifies three higher-order categories of gambling motivations (self-determined, non-self-determined, and amotivation) covering seven distinct factors (intrinsic-knowledge, intrinsic-accomplishment, intrinsic-stimulation, extrinsic-identified regulation, extrinsic-external regulation, extrinsic-introjected regulation, and amotivation). Using this scale, Oei and Raylu (2010) found that Chinese gamblers in Australia had higher motivation in the extrinsic-identified regulation domain compared to European-origin gamblers, who had higher levels of the intrinsic-stimulation motivation. This indicates that while people who identify as of European-origin tend to be motivated to gamble for excitement, those who identify as Chinese often attach greater meaning to gambling, using gambling as a social outlet or way to gain social status and prestige in the eyes of others (Victorian Casino and Gaming Authority, 2000).

Supporting the idea that gambling can take on meaning beyond that of a recreational activity, one of the few qualitative studies in this area focused on casino gamblers in Las Vegas and attempted to categorise culturally diverse gamblers by ‘type’. Distinct differences between cultural groups were identified (Latour et al., 2009). Chinese gamblers were given the type-label ‘Distinction’, wherein gambling was viewed as a risk-taking activity instinctually tied to self-worth, as reflected in luck and the ability to acquire wealth quickly. Other research has supported these findings. In addition to social motivations, the desire to acquire wealth quickly is a particularly strong motivator among East Asian gamblers (Chan et al., 2015; Lam, 2005).

## Gambling Activity Preferences

Differences in gambling activity preferences across different cultural groups have also been identified. Much research has been conducted into the relative danger and harm of EGMs, which are generally agreed to be the most problematic gambling activity (Delfabbro et al., 2020). In contrast, Welte et al. (2007) found that casino gambling is responsible for more problems per day of play than other forms of gambling but less responsible overall due to lower engagement with this activity. In Australia, approximately 15% of people who play casino table games are problem gamblers, compared to approximately 6% of EGM players (Armstrong & Carroll, 2017). Generally, problem gamblers tend to gamble across a wide range of activities (Armstrong & Carroll, 2017). However, there is empirical and anecdotal evidence that casino table games are a particular preference for those of East Asian descent, with high rates of participation in this activity consistently found when compared to other ethnicities (Liu & Wan, 2011; Williams et al., 2021). Although it is now relatively old research, Fisher (2000) found that in the United Kingdom, problem gambler casino patrons who regularly gambled at the casino were more likely to come from an ethnic minority group compared to non-regular casino patrons, with 73% of these ethnic minority gamblers being of Asian descent.

Table games also provide the opportunity to win large sums quickly and, as previous evidence has found, rapid acquirement of wealth is a strong motivation to gamble (Lam, 2005). This is likely a particular attraction of the casino environment compared to local clubs and hotels. However, the availability of table games at casinos is not necessarily the only factor that makes these venues appealing. Research has found that, while EGM participation by some Asian gamblers is lower in clubs and hotels compared to other ethnicities, Asian gamblers often engage with EGMs at a similar rate to other ethnicities in a casino environment (Victorian Casino and Gaming Authority, 2000). This indicates a more complex interaction between the venue and gambling activity that requires further research.

## Help-Seeking and Help-Seeking Barriers

### Known Patterns in Help-Seeking

Gambling research designed to inform policy has largely shifted away from placing responsibility for gambling problems within the personal purview of the gambler. Instead, it has shifted towards a public health approach that views gambling problems as the social responsibility of gambling operators and regulators (Hing, 2002). Despite this, many major harm minimisation strategies do not reflect this shift and largely rely on gamblers initiating engagement with advertised gambling help services and other responsible gambling initiatives of their own accord (Francis & Livingstone, 2021). In general, approximately 1–2% of all gamblers seek help for gambling-related problems (Australian Institute of Health and Welfare, 2021; Menzies School of Health Research, 2019). However, this percentage is heavily influenced by the larger proportion of gamblers in the population who do not experience significant gambling problems and whose help-seeking rates are low (Department of Justice and Attorney-General, 2018). Compared to gamblers in general, help-seeking rates are much higher among problem gamblers, at approximately 20% in 2016-17 (Department of Justice and Attorney-General, 2018).

Given the differences in motivations to gamble, gambling behaviours, and the high prevalence of problem gambling among Asian gamblers, this group is particularly vulnerable to gambling-related harms, which should be reflected in help service utilisation data. However, currently unpublished analysis of government help service data by the authors of this paper indicates that most help service clients in Australia identify as Australian or as being born in Australia. There is limited information on the uptake of gambling help services by CALD gamblers in Australia. Some older research indicated low engagement with help services (McMillen et al., 2004; Victorian Casino and Gaming Authority, 2000), which is inconsistent with current international trends. For example, data from New Zealand indicates that help service utilisation among Asian ethnicities closely aligns with the ethnicity distribution in the general population (Ministry of Health, 2021). However, the ‘Asian’ group in that dataset is a wide classification containing a large number of ethnicities.

Gambling-related data in Australia is regularly collected through a range of extensive surveys conducted at national, state, and territory levels. A major survey conducted every five years in Queensland is the Queensland Household Gambling Survey (QHGS), which surveyed 15,000 Queensland residents in its latest iteration. In 2018, the QHGS found that, in general, Queenslanders would turn first to Gambling Helpline, then Gamblers Anonymous, followed by family and friends for help with gambling problems (Department of Justice and Attorney-General, 2018). However, rather than turning first to government-funded services, previous studies consistently indicate that East Asian gamblers will first seek help from extended family and close community (John & Williams, 2013; McMillen et al., 2004; Tse et al., 2007; Victorian Casino and Gaming Authority, 2000).

Asian gamblers may have different views of what problems warrant seeking professional help. For example, Chinese gamblers may not see gambling problems as something that requires the intervention of a counsellor. There is often the belief that counsellors are consulted about things that are ‘bad’, whereas gambling is seen as a reflection of a positive belief in luck (McMillen et al., 2004). Further, how gambling is viewed within some East Asian cultural communities can lead to difficulty in acknowledging when acceptable gambling has transitioned into problematic gambling (Wong & Tse, 2003). When gambling is viewed through the lens of being a social activity and associated with cultural ideals of luck and prosperity, it can be difficult to interpret it as problematic.

There may also be a difference in the time gap between the onset of gambling and treatment seeking for problem gambling, which is generally quite long. In a study of Chinese gamblers in Singapore, on average, it took approximately 20 years for participants to seek treatment for their gambling problems, with many not seeking help at all (Teo et al., 2007). This partly reflects the time it may take for a gambling problem to develop and delays in receiving help due to the resource limitations of services (Kaufman et al., 2017). However, it could also indicate some delay between needing help and seeking help.

Anecdotal evidence from discussions with CALD help services identified in the stakeholder scan supports previous research findings (Victorian Casino and Gaming Authority, 2000) that the limited uptake of gambling help services does not necessarily reflect the degree of gambling problems within the community. Help service providers have indicated that gamblers often access other services such as financial assistance, food assistance, and relationship counselling without disclosing problems with gambling. These issues are often not unveiled until service providers identify patterns in assistance-seeking that are suggestive of gambling problems (Carroll et al., 2011). While discrete evidence for this is lacking in the context of CALD gamblers specifically, it is reasonable to assume that similar patterns exist within this group, especially considering the established reluctance among CALD community members to disclose gambling-related problems (Scull & Woolcock, 2005). Further research is needed to determine if CALD gamblers are accessing help services at a rate that reflects what is known about the prevalence of problem gambling within CALD communities.

### Known Barriers to Seeking Help

There is evidence that cultural background can have an impact on the accessibility of help services. Identified barriers include suspicion of mainstream services, concerns about confidentiality, language barriers, lack of cultural safety, experiences of discrimination, unfamiliarity with the concept of Western counselling services, fear of stigma within the community, feelings of shame, and fear of loss of face (Abbato, 2011; McMillen et al., 2004). Shame and loss of face are particularly pertinent barriers to help-seeking for gamblers situated within collectivist cultures. These factors negatively impact the entire family unit rather than solely the individual (Abbato, 2011; Radermacher et al., 2016). This leads to reluctance to expose any existing problems (Scull & Woolcock, 2005), especially as people of East Asian descent report higher levels of stigmatisation towards problem gambling than those who identify as of European-origin (Dhillon et al., 2011). It remains unclear whether these specific barriers experienced by CALD gamblers affect gamblers with different Asian cultural orientations in Australia and Asian-born Australians to the same extent (Tata & Leong, 1994; Yang et al., 2007). This is another avenue for future research.

When help is sought, evidence suggests services are not necessarily culturally appropriate. Mainstream services are often not designed to align with non-White, westernised cultural systems and values. For example, in some cultures, it may be inappropriate for a woman to have a male counsellor, or the inclusion of family members may be an important intervention component (Seah et al., 2002). Western-influenced help services are often not designed to adapt to these cultural needs and may not be equipped to provide culturally appropriate help for some CALD clients. For example, there is evidence that this limitation in service delivery is a barrier to formal help-seeking among Indigenous Australians with gambling problems (Jurisic, 2011; McMillen et al., 2004). Hing et al. (2014) found that while some Indigenous Australians still utilised formal help services, 29% of Indigenous Australian respondents classified as problem gamblers (12% across all gambling risk groups) reported not seeking help because they thought services would not understand their cultural background. While reluctance to seek help is certainly not confined to CALD gamblers (Itapuisto, 2019; Pulford et al., 2008), it is reasonable to assume that these kinds of barriers to help-seeking are particular to those who experience broader marginalisation, such as CALD gamblers, and would likely not be experienced by those who belong to the dominant cultural group in Australia for whom help service providers largely design their programs.

The stakeholder scan conducted as part of this review identified many publicly available government-funded gambling help services that offer multi-lingual support to be accessible to CALD gamblers (See Table A1 in Supplementary materials). These services include Gambling Help Online, Gambling Help Service, Gambling Help Queensland, and various charitable and religious bodies such as St Vincent de Paul and the Wesley Mission. However, it is likely that simply offering translation options is insufficient to address the cultural barriers encountered (Malatzky et al., 2018).

The stakeholder scan also identified several other services, particularly community groups, that may be better positioned to provide culturally appropriate assistance to CALD gamblers. Similar to mainstream help services, assistance is commonly offered in the form of counselling or educational programs for individual gamblers and their families. However, these services are relatively limited in number, and many do not have the same degree of resources found in larger mainstream gambling help services. The stakeholder scan found that culturally tailored gambling help services are generally one of several social support services offered by community groups rather than organisations dedicated solely to gambling help. Further, most of these services are provided by groups that service clients of either Chinese or Vietnamese heritage. For example, the Australian Vietnamese Women’s Association offers a gambling counselling service, EACH provides the Chinese Peer Support Program, and Community Access and Services South Australia has the Vietnamese Gambling Help Service. While certainly useful, the narrow scope of these help services may mean that there are gamblers from other CALD groups who do not have access to culturally appropriate support services. Directing resources towards providing culturally appropriate, and thus effective, services is important to ensure help can be readily accessed by CALD community members.

## Methodological Limitations in and Opportunities for Gambling Research

A review of the literature provides strong evidence for gambling problems within CALD communities and in gamblers either from or raised within families from East Asia. However, differences exist in how ethnicity and cultural groups are defined. The nature of ethnicity is multidimensional and can include elements such as race, ancestry, nationality, religion, language, and country of birth, as well as culture (Burton et al., 2010). The major surveys that collect gambling data in Australia use various definitions and measurements of ethnicity to make between-group comparisons and examine cultural differences. For example, the QHGS simply asks participants if they were born in Australia or ‘other’, with an option for ‘don’t know’ (Department of Justice and Attorney-General, 2018). These response options are limiting as only having data about the country of birth does not allow for more granulated information about cultural orientations, backgrounds, or ethnicity to be considered.

Another Australian government survey, The Victorian Population Gambling and Health Study (Rockloff et al., 2020), utilised speaking a language other than English at home to inform group comparisons. While this provides more information than responses to country of birth, it also has the potential for misclassification, as speaking a language other than English at home does not necessarily mean that the respondent identifies with the culture or ethnicity associated with that linguistic background, especially in multi-generational households. An alternative approach has been to ask respondents which ethnicity they most strongly identify with. This approach considers multicultural backgrounds and allows the respondent to select an ethnic background that they feel is most accurate. However, this still has the potential to limit responses and relies on respondents understanding nuances between the concepts of race, ethnicity, and nationality in the same manner as the researcher, and therefore may not ultimately reflect the respondents’ cultural identification.

Any self-report survey that provides response options for ‘race’, culture, or ethnicity will be influenced by how respondents understand nuances around these concepts, how well they identify with the options provided (Bonnett & Carrington, 2000), and the researchers providing comprehensive response options relevant to the population being sampled. This may become a source of measurement error in surveys due to respondents and researchers using these terms interchangeably (Ford & Kelly, 2005). In other instances, respondents may not be given the opportunity to differentiate between these concepts or not be given sufficient options from which to select (Burton et al., 2010; Eisenhower et al., 2014). In contrast, giving respondents the freedom to answer questions about ethnicity via open-ended questions can introduce substantial variability and inconsistency compared to selecting from a range of pre-defined categories, affecting the accuracy of between-group comparisons (Lopez, 2003).

Sampling procedures and assessment of ethnicity must be carefully considered in the construction of gambling surveys to ensure that respondents are accurately represented. Utilising multidimensional measures of collecting cultural data, comprised of multiple questions attempting to differentiate between culture, ethnicity, country of origin, and country of parents’ birth, can provide the most accurate picture (Parameshwaran & Engzell, 2015; Williams & Husk, 2013). There is evidence that ethnic minorities, including people of East Asian descent, are often underrepresented in wider research due to a combination of factors, including language barriers, reluctance to disclose personal information and a lower likelihood of being invited to participate (Sheikh, 2006; Smart & Harrison, 2017; Teo et al., 2017). As problem gamblers are proportionately rare in the community, ensuring that all cultural groups are accurately captured can present a substantial challenge despite the use of statistical methods such as data weighting. There is anecdotal evidence that reluctance to participate in surveys is not only the case for large surveys conducted by government or research organisations; it is also found in customer surveys initiated by gambling operators. This can have significant repercussions if the conclusions drawn from non-representative cross-cultural comparisons are used to inform policy and resource distribution.

It is also important to note that a substantial proportion of the gambling studies in this review utilise quantitative study designs, often in the form of self-report questionnaires. Qualitative forms of inquiry enable researchers to develop nuanced, culturally-situated understandings of gambling-related phenomena. While underreporting and social desirability bias may still occur in qualitative research (Adams et al., 2005; Holtgraves, 2004; Krumpal, 2011; Tourangeau & Yan, 2007), qualitative methods of data collection allow researchers to employ several techniques, such as indirect questioning and requesting stories or examples that can help limit social desirability responses (Bergen & Labonté, 2020), and provide richer, more complex data. Insights into the lived experience of gamblers and the impacts of gambling and casinos within CALD communities generated through qualitative research could help guide and advance the field of gambling studies in several related directions.

Gambling research would also benefit from a greater emphasis on assessing gambling-related benefits. While there has been much investigation into gambling-related harms in the general community, there has been little assessment of the benefits gamblers derive from their gambling involvement. Cultural variations in these benefits have received even less attention. Most of the literature relating to the benefits of gambling focuses on the economy or tourism (Chhabra, 2007; Farrow, 2015; Walker, 2007) or is limited to perceived benefits among narrow samples such as college students or people with a disability (Pitt et al., 2020; Wickwire et al., 2007). Responsible gambling initiatives attempt to balance consumer freedoms and the benefits communities and individuals derive from gambling against the established harms (Basham & Luik, 2011; Hing, 2010). However, for harm minimisation policy to be informed by a balanced assessment of the impacts of gambling, there must be a substantial increase in the focus given to the social, psychological, and financial benefits of gambling experienced by the gambler.

## Conclusion and Future Directions

Gambling-related harm is likely to continue to be an issue at the forefront of Australian public health research, especially as gambling availability continues to increase. With increases in gambling availability, there is likely to be an increase in harm. Research informing how best to mitigate this harm should engage with those who experience greater vulnerability. Existing research provides strong evidence about cultural variations in gambling motivations, beliefs, behaviours, harms, and help service utilisation. However, much of this research is relatively old and may not reflect contemporary changes in Australia’s gambling environment and cultural profile. Previous research on East Asian gamblers has mainly focused on individual cultural groups within their country of origin, international students, or migrants from a limited selection of Asian countries. There is a current lack of research focusing on Australian gamblers of East Asian descent who have been raised with a combination of Eastern and Western cultural influences. Their experiences of gambling and its impacts may be different to Asian gamblers born overseas. Additionally, it is unclear how Asian-born Australians with first-generation immigrant families identify their ethnicity in surveys. This limitation in the collection of demographic information makes determining the experiences of this group difficult.

New research that collects ethnic demographic data in more detail and samples CALD gamblers more extensively is needed. Further, the experiences and circumstances of casino gamblers appear to differ from those of other gamblers. There is a lack of contemporary research focused on casino gamblers from CALD groups in Australia. As East Asian gamblers indicate a preference for casino table games, studying this group, in particular, will provide insight into the effect of casino gambling on those vulnerable to harm from this type of venue.

Considering the evidence of high levels of problematic gambling among some CALD gamblers, ensuring that accessible supports are in place is a critical component of harm minimisation. Previous studies of general barriers to help service utilisation for CALD gamblers provide evidence pointing to utilising family, friends, and community services as avenues of support once crisis situations have been reached. However, little is known about the effectiveness of these avenues of help or the current uptake of formal help services among this group. Combining quantitative methods with qualitative forms of inquiry would be an ideal avenue for future research on these topics. The new understandings produced through such research are needed to better inform policy and ensure that government funding and responsible gambling resources are directed towards services that will be the most accessible and effective for diverse CALD communities.

## Electronic Supplementary Material

Below is the link to the electronic supplementary material.

Supplementary Material 1

## Data Availability

All data generated or analysed during this study are included in this published article [and its supplementary information files].

## Declarations

All authors contributed to the article conception and design. The stakeholder scan was performed by Thuy Vi Doan. The literature search was conducted by Victoria Rowlatt and Thuy Vi Doan, and the literature was synthesised by Victoria Rowlatt. The first draft of the manuscript was written by Victoria Rowlatt and all authors commented on previous versions of the manuscript. Darren Wraith and Christina Malatzky provided supervision, editing, and critical review on all versions of the manuscript. All authors read and approved the final manuscript.
