# Supplementary material for: Culturally and Linguistically Diverse Gamblers of East Asian Descent in Australia: A Comprehensive Review of Current Evidence
Source: J Gambl Stud. 2023 Mar 28;39(2):947–69. doi: 10.1007/s10899-023-10202-5 (PMC10175356; doi:10.1007/s10899-023-10202-5)
Supplement: Supplementary file 1 — Supplementary Material 1 [file 10899_2023_10202_MOESM1_ESM.docx]

**Culturally and Linguistically Diverse Gamblers of Asian Descent in Australia: A Comprehensive Review of Current Evidence**

**Supplementary Materials**

Table A1

| **Organisation** | **Organisation Description** | **Gambling-Related Help Services** | **Cultural Affiliation** | **Region Serviced** | **Funding** |
| --- | --- | --- | --- | --- | --- |
| **Australian Vietnamese Women’s Association (AVWA)**  https://www.avwa.org.au/en/services/health-community-well-being/gambling-counselling | AVWA is a non-for-profit organisation that provides settlement and integration assistance for refugees and migrants. AVWA also offers a range of individual advocacy work to improve the quality of life for individuals from CALD communities living in Australia. | The gambling counselling service is part of the AVWA Rehabilitation, Health and Wellbeing program. It includes face-to-face, phone or email counselling services to all Vietnamese Victorians experiencing problem gambling both directly and indirectly. There is also a prisoner support program for Vietnamese individuals and their families. | Vietnamese | Victoria | Funded by the Victorian Responsible Gambling Foundation |
| **Community Access and Services South Australia (CAaSSA)**  https://www.caassa.org.au/gambling-help/ | CAaSSA is a not-for-profit organisation that supports CALD communities in Northern and Western regions of South Australia. General programs and assistance include aged care, addiction services, community participation activities, the NDIS, mental health and youth support, community education and cultural connection. | Vietnamese Gambling Help Service (VGHS) is accessible to individuals with a Vietnamese background who are impacted by problem gambling and those who are indirectly affected. This service offers individualised support, gambling therapy and community education. | CAaSSA – CALD Groups  VGHS – Vietnamese | South Australia | Funded by the South Australian Department of Human Services through the Office of Problem Gambling. |
| **EACH**  https://www.each.com.au/service/chinese-peer-connection/ | EACH delivers health, disability, counselling and mental health services nationwide. It focuses on social and economic factors negatively affecting the health and wellbeing of individuals. | The Chinese Peer Support Program (CPSP) is a free telephone service that aims to support individuals from the Chinese community who experience gambling-related harm through trained peer support, counselling or group work facilitated by those who have previous experience with problem gambling. The program also extends to family and friends. | Chinese | Nationwide | EACH is funded by the Australian Government, the Victorian State Government (including the Victorian Responsible Gambling Foundation) and NSW Government. |
| **Federation of Chinese Associations Inc.**  https://www.vicfca.org.au/vicfcagroup | The Federation of Chinese Associations is a non-profit, non-religious coalition of Chinese communities. The state chapters are community organisations without any specific gambling-related focus. | In Victoria, the Federation of Chinese Associations (VIC) Inc provide the Chinese Aid Centre (formerly known as the Victorian Chinese Federation Welfare Centre), which offers various social work services. The centre offers healthy lifestyle services, which include counsellors for Chinese gamblers. | Chinese | Nationwide, with state chapters | No publicly available funding information. |
| **Chinese Gambling Concern Inc.**  http://cgci.org.au/ | CGCI is a non-profit organisation founded in 1996 by professional counsellors and social workers concerned about the adverse impact of gambling that they had observed in the ethnic Chinese community. The objective of CGCI is to educate about the potential harms of gambling and provide support to those negatively affected. | CGCI is a dedicated gambling-related help service that supports people of Chinese ethnicity experiencing negative effects due to gambling. They have several services, including a free helpline, professional holistically based counselling service, educational programs, assistance with self-exclusion and pre-commitment schemes, and assistance with referrals to other help services. CGCI also holds several social events designed to raise awareness of gambling risks for the Victorian Chinese community. | Chinese | Victoria | Receives funding from the Victorian Responsible Gambling Foundation. |
| **Springvale Indochinese Mutual Assistance Association (SICMAA)**  https://sicmaa.com/about-2/ | SICMAA is a not-for-profit organisation that enables social and economic opportunities for Vietnamese people in the community, provides advocacy work and counselling services, and assists with acculturation processes. | SICMAA provides limited financial assistance such as emergency relief payments, family support, particularly with family violence and parental relationships, recreational activities and an in-language counselling program for Vietnamese people affected by gambling (Hope Program). | Vietnamese | Victoria | No publicly available funding information. |
| **Vietnamese Community in Australia – NSW Chapter Inc.**  **Vietnamese Community in Australia – QLD Chapter Inc.**  https://vcaqld.org.au/index.php/en/ | The Vietnamese Community Chapter Inc is established across Australia. The not-for-profit community organisations in various states represent and support Vietnamese immigrants and Vietnamese Australians. | The chapters of Vietnamese Community in Australia do not offer any official gambling-related services; however, they offer general support to the Vietnamese community across various social issues, including emergency relief support and individual case management. | Vietnamese | Nationwide, with chapters in various States | The Queensland Chapter receives funding from the Queensland Government, which is supplemented by various fundraising efforts by the organisation. |
| **GambleAware**  https://www.gambleaware.nsw.gov.au/ | GambleAware aims to work towards “zero” gambling-related harm in New South Wales through research, education and support for individuals and communities. | GambleAware intends to promote safe and responsible gambling and prevent gambling-related impacts. These main goals are carried out by providing clinical support services, advancing research and public health approaches such as offering educational materials and providing opportunities and resources to enable communities to overall reduce the negative effects of gambling. | Non-specific | New South Wales | Funded by the NSW Responsible Gambling Fund. |
| **Connect Health & Community**  https://connecthealth.org.au/preventing-gambling-harm/ | Connect Health & Community is a not-for-profit community health service assisting residents across various health, mental health and social support services. | Connect Health & Community has run The Gambler’s Help Southern program (in association with Gambler’s Help) since 1995, offering free and confidential gambling help services to Melbourne residents, including counselling (financial and therapeutic), venue support, community engagement and school education. The service works with a range of interpreters to provide help in multiple languages. | Non-specific | Melbourne, Victoria. | Funded through Commonwealth, state and Local Government grants. |
| **Gamblers Anonymous (GA)**  https://gaaustralia.org.au/home-about/ | Gamblers Anonymous is an organisation that provides a space for individuals who are experiencing and/or are recovering from problem gambling to share their experience and gain strength and hope from others. | GA hosts approximately 130 meetings nationwide. These meetings enable individuals affected by gambling-related harm to share their difficulties and heal in a safe environment. | Non-specific | Nationwide | Self-funded |
| **Gambling Help Queensland (GHQ)**  https://www.gamblinghelpqld.org.au/ | GHQ is a free service available to the public. | The helpline and counselling services are formed and delivered by a network of community-based specialist support agencies to individuals and affected others. GHQ also delivers community education and training and is involved in community network events, to promote and enhance community understanding and awareness. | Non-specific | Queensland | Funded by the Queensland Government. |
| **Gamblers Help**  https://gamblershelp.com.au/ | Gamblers Help, Victoria, offers both telephone and face-to-face support services for those negatively affected by gambling. Gamblers Help also in | Services include phone support, Peer Connection, face-to-face counselling, online support and financial counselling. Counselling services are provided in various languages and assists with organising interpreter services.  Gamblers Help also offers the Chinese Peer Connection Program, designed to assist Chinese gamblers and their family members with support in a non-crisis situation. | Non-specific (general services)  Chinese (Chinese Peer Connection program) | Victoria | Government-funded via the Responsible Gambling Fund, administered by the Victorian Responsible Gambling Foundation. |
| **Gambling Help Online**  https://www.gamblinghelponline.org.au/ | Gambling Help Online is a service designed to assist in providing help for gambling problems to people who are reluctant or unable to access face-to-face services. | Gambling Help Online offers free 24/7 counselling and support services via live chat and email. The website also offers an extensive range of educational materials, self-help information and an online forum for peer support. | Non-specific | National | Funded by state and National governments. |
| **Oakdene House Foundation**  https://www.oakdenehouse.org.au/services/ | Oakdene House Foundation assists individuals experiencing the impacts of problem gambling or alcoholism. The non-profit organisation also offers support to family and friends. | Oakdene House Foundation provides support and guidance for addicts, families and significant others, including counselling services, suicide prevention counselling, short-term assessment and recovery preparation for sufferers of addiction. The Foundation also provides financial counselling, Gamblers/Alcoholics Anonymous meetings and educational presentations.  Oakdene House Foundation runs the Life Choices Program (a 6-week group educational program). Wesley Mission provides responsible Gambling Counselling. | Non-specific | New South Whales | No publicly available funding information. |
| **Relationships Australia Queensland (RAQ)**  https://www.raq.org.au/services/gambling-help-program-ghs | Relationships Australia Queensland promotes respect, and fair and safe relationships, especially among groups and communities at greater risk or disadvantage. | The Gambling Help Program (GHS) consists of gambling and financial counselling, community education and interventions, self-exclusion support from gambling sites and conducting referrals. Counselling can occur either face-to-face or over the phone. Support for those who speak a language other than English is available in conjunction with the aid of a free interpreter. | Non-specific | Queensland (also nationwide chapters) | Government-funded at federal, state, and local government levels. |
| **St Vincent de Paul Society Queensland (St Vinnies QLD)**  https://qld.vinnies.org.au/who-we-help/drug-alcohol-gambling-services | St Vincent de Paul Society Queensland supports individuals and communities in various ways. The types and levels of assistance target population groups such as children and families, people in need of housing, migrants and refugees, people experiencing addiction and more. | Drug, alcohol and gambling services at St Vinnies QLD apply holistic approaches to address an individual’s addiction. In Queensland, people affected by gambling can access support centres and call centres via the helpline.  Support services that the St Vincent de Paul Society offers include face-to-face counselling, referrals, and support to family members. The services are free of charge for people with gambling problems and their families. Services vary by state. | Non-specific | Queensland (also nationwide chapters) | St Vincent de Paul receives government funding for some of the Society’s Special Works, with the rest of the services carried out by members and volunteers. |
| **Wesley Mission Queensland**  https://www.wmq.org.au/mental-health/wellbeing-mental-health-service | Wesley Mission Queensland aims to offer care and compassion whilst promoting individual choice, independence and collective community wellbeing by providing various services. These include disability and NDIS support, parenting programs, crisis relief, housing and homelessness assistance, and health, mental health and wellbeing services. | Wellbeing Mental Health Service offers free, short-term therapeutic support (between four to six sessions) for individuals living in Brisbane, South region. This service can be beneficial for those wanting to address alcohol and other drugs, gambling, or other addictions through participating in peer support/guidance, coaching, education or receiving practical assistance, coping strategies and life skills. | Non-specific | Queensland (also nationwide chapters) | Wesley Community Services is registered as a Public Benevolent Institution and does receive some government funding. |
